# Modelling the effects of media during an influenza epidemic

**DOI:** 10.1186/1471-2458-14-376

**Published:** 2014-04-17

**Authors:** Shannon Collinson, Jane M Heffernan

**Affiliations:** 1 Department of Mathematics & Statistics, York University, Toronto, Canada; 2 Modelling Infection and Immunity Lab, Centre for Disease Modelling, York Institute for Health Research, York University, Toronto, Canada

**Keywords:** Mass media, Epidemic, Influenza, Agent-based Monte Carlo simulation

## Abstract

**Background:**

Mass media is used to inform individuals regarding diseases within a population. The effects of mass media during disease outbreaks have been studied in the mathematical modelling literature, by including ‘media functions’ that affect transmission rates in mathematical epidemiological models. The choice of function to employ, however, varies, and thus, epidemic outcomes that are important to inform public health may be affected.

**Methods:**

We present a survey of the disease modelling literature with the effects of mass media. We present a comparison of the functions employed and compare epidemic results parameterized for an influenza outbreak. An agent-based Monte Carlo simulation is created to access variability around key epidemic measurements, and a sensitivity analysis is completed in order to gain insight into which model parameters have the largest influence on epidemic outcomes.

**Results:**

Epidemic outcome depends on the media function chosen. Parameters that most influence key epidemic outcomes are different for each media function.

**Conclusion:**

Different functions used to represent the effects of media during an epidemic will affect the outcomes of a disease model, including the variability in key epidemic measurements. Thus, media functions may not best represent the effects of media during an epidemic. A new method for modelling the effects of media needs to be considered.

## Background

Influenza causes annual epidemics and occasional pandemics, which have claimed millions of lives throughout history. In the past century four worldwide pandemic outbreaks of influenza have occurred: 1918, 1957, 1977 and 2009, [1,2]. According to the Public Health Agency of Canada, inter-pandemic (or seasonal) influenza affects approximately 20,000 Canadians, with approximately 2,000 to 8,000 deaths annually [3]. In the USA, it has been reported that flu-associated deaths can range from 3,000 to 49,000 individuals per year [4].

Mass media can affect disease transmission during an influenza epidemic or pandemic. Attention to health news has been increasing in importance during the last few decades, and thus, media reports can play an important role in defining health issues, since they serve as a major source of information and are able to incite changes in behaviour in the public [5]. Individual response to a disease threat depends on risk perception that is gained largely through information reported by governments to mass media: number of infections, hospitalizations and deaths, as provided by the government [6,7].

We have recently seen the use of mass media reports during two infectious disease outbreaks. The first novel infectious disease of the twenty-first century was SARS. It had distinct features such as rapid spatial spread and self-control, and vast media coverage [6,7] that used to inform the public, provide a means of contract tracing, and advise isolation of exposed individuals.

Media coverage was substantial during the H1N1 epidemic in 2009 as well, which may have had an effect on the transmission of influenza by promoting social distancing and self-isolation [8]. The media coverage of this influenza pandemic was widespread, with an increased sense of urgency since this influenza strain was related to the 1918 pandemic strain that caused approximately 50 million deaths worldwide [1].

Mathematical modelling has been used to study the effect of mass media on epidemics by employing the well-known Susceptible-Exposed-Infectious-Recovered (SEIR) model and various extensions [6-14]. In these studies, mass media has been incorporated using different, but qualitatively similar, functions that directly affect disease transmission and susceptibility. In general, the chosen functions are decreasing functions with respect to the current number of infected individuals in the population. However, the choice of function seems to be arbitrary. It is possible that the choice of function can change study results. For example, public health officials could be interested in epidemic measurements such as the peak number of infection, peak time, total number of infections and end of epidemic, which are all directly related to important public health resources (i.e. number of hospital bed, antiviral stockpile, vaccination doses). These key measurement may vary depending on the chosen media function. Therefore, a sensitivity analysis on these functions is required.

In addition to that mentioned above, there is a further drawback of the previous studies which include media functions, in that, deterministic systems of ordinary differential equations are employed. Deterministic models can describe the mean behaviour of an epidemic, but information surrounding any variability in key epidemic measurements cannot be made. A stochastic model is well suited to this task. Various methods of introducing stochasticity into disease models exist: [15-19]. Agent-Based Monte Carlo (ABMC) simulations, provide a way in which individuals with certain disease characteristics can be tracked in a population. This method also provides flexibility, as changes in biological assumptions can be easily incorporated, which are difficult to include in other methods.

In the sections that follow we give an overview of the functions used to describe media in the disease modelling literature. The functions are then incorporated into a standardized SEIR model, and model results are compared. A stochastic agent-based Monte Carlo (ABMC) simulation is then introduced, and is employed to study the variability within an epidemic depending on the media function chosen. A sensitivity analysis is also completed in order to determine the importance of certain model parameters on various epidemic outcomes for each media function.

## Methods

### Media functions

From the disease modelling literature [6-14] we have identified three distinct functions employed to present the effects of mass media:

(1) $$\begin{array}{l} f\left(I,p_{1}\right)=e^{-p_{1}\gamma I} \end{array}$$

(2) $$\begin{array}{l} f\left(I,p_{2}\right)=\left(\frac{1}{1+p_{2}I^{2}}\right) \end{array}$$

(3) $$\begin{array}{l} f\left(I,p_{3}\right)=\left(1-\frac{I}{p_{3}+I}\right) \end{array}$$

where *I* is the number of infectious individuals in a population, *γ* is the recovery rate, and *p*_*i*_, *i* = 1, 2, 3 is a parameter used to represent media effects in these functions. In general, these functions are decreasing functions of *I*, which represents the fact that, as the number of infections increases in a population, and is reported by mass media, susceptible individuals will practice social distancing or control measures, which decreases susceptibility. Comparing the three functions, we see that, for a given *I* and *γ*, *p*_1_ and *p*_2_ can be written in terms of *p*_3_:

(4) $$\begin{array}{l} p_{1}=\frac{-\ln\left(\frac{p_{3}}{p_{3}+I_{c}}\right)}{\gamma I_{c}} \end{array}$$

(5) $$\begin{array}{l} p_{2}=\frac{1}{p_{3}I_{c}}\;. \end{array}$$

These equations demonstrate that, if *p*_3_ is increased, then *p*_1_ and *p*_2_ must decrease to have the functions remain the same value at a chosen *I*_*c*_.

**Figure 1:**
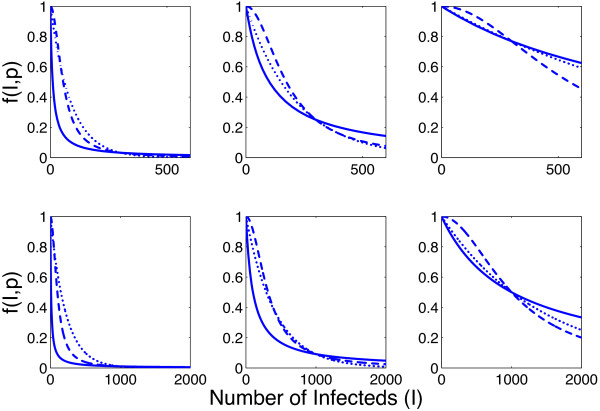
**Media Functions** ***f*(*I*,*p*) for different values of** ***p*.** Functions (1), (2) and (3) (dotted, dashed, solid lines respectively) are shown when *p*_3_ = 10 (left), 100 (middle), 1000 (right), *γ* = 1/4 and *I*_*c*_ = 300 (top), 1000 (bottom). *p*_1_ and *p*_2_ are calculated using equations (4) and (5).

In Figure 1, we plot Functions (1)-(3) (dotted, dashed, solid lines respectively) for media parameters *p*_*i*_, *i* = 1, 2, 3 (dotted, dashed, solid lines) with *γ* = 1/4 and *I*_*c*_ = 300 (top) and *I*_*c*_ = 1000 (bottom). Note that the functions are equal when *I* = 300 (top) and 1000 (bottom). Here, *p*_3_ = 10 (left panel), 100 (middle panel), and 1000 (right panel), and *p*1 and *p*2 are determined using Equations (4) and (5).

### SEIR framework

To compare the effects of different media Functions (1)-(3), we must choose a standardized model. For the purposes of our study, we have chosen the basic Susceptible-Exposed-Infectious-Recovered (SEIR) model with a constant population size *N*:

(6) $$\begin{array}{l} \dot{S}=-f(I,p)\beta \text{SI}\\ \dot{E}=f(I,p)\beta \text{SI}-\sigma E\\ \;\dot{I}=\sigma E-\gamma I\\ \dot{R}=\gamma I\\ N=S+E+I+R\;. \end{array}$$

Briefly, susceptible individuals (*S*) are infected by infectious individuals (*I*) with rate *β* and become exposed (*E*). Transmission can also be reduced as determined by *f*(*I*,*p*). Exposed individuals become infectious with rate *σ*, and infectious individuals recover with rate *γ*. The initial conditions and parameters values for this study can be found in Table 1.

**Table 1:** Initial conditions and parameter values for model (6), functions (1)-(3) and the ABMC

	**Description**	**Value**	**Range**	**Unit**	**Reference**
Population					
S	Susceptible	10,000			
E	Exposed	0			
I	Infectious	10			
R	Recovered	0			
Parameter					
*R*_0_	Basic reproductive ratio	1.5	1.3-1.7		[20,21]
*β*	Contact transmission rate	3.71287*e*-5		(person-day) ^-1^	Eq. (7)
*σ*	Transition rate *E* to *I*	1/2		(day) ^-1^	[20]
*γ*	Recovery rate	1/4		(day) ^-1^	[20]
*p*_*i*_	Media parameter	varies	varies		[7]
ABMC					
*S*/*β*	Mean time to transmission	*S*(*t*)/*β*			Eq. (7)
1/*σ*	Mean exposed time	2		days	[20]
1/*γ*	Mean infectious time	4		days	[20]
1/*p*_*i*_	Media parameter	varies	varies		[7]

### Agent-based Monte Carlo simulation

**Figure 2:**
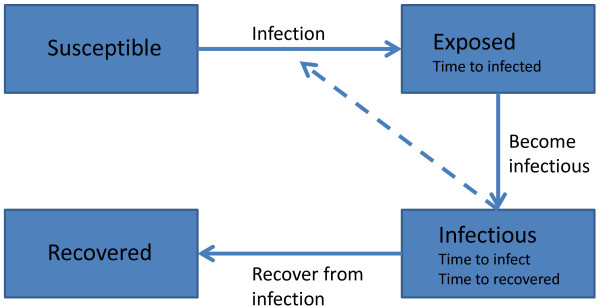
**Schematic of the Agent Based Monte Carlo simulations corresponding to Table**1**.**

For further comparison of the SEIR model with different media functions, we utilize an Agent-Based Monte Carlo (ABMC) simulation to capture the variability in the epidemic infection curve (that cannot be determined using a system of deterministic equations like that of System (6)). There are various ways of developing an ABMC simulation. We have employed a previous method as developed by Heffernan and Wahl, [16,22]. Briefly, the ABMC simulation moves forward in time following event times: the next time that an individual changes state within the system. Agents in each of the susceptible, exposed, infectious and recovered compartments are assigned event times corresponding to Table 1 when they are introduced into the simulation, for each event that allows such an individual from that compartment to change state. For example, exposed individuals are assigned a time to become infectious, and infectious individuals are assigned a time to recovery and a time to infect a susceptible. The minimum event time determines the next event to occur in the simulation. The event is then carried out, and the next event is determined. To compare to the SEIR system as described above, we assume exponential distributions for all parameters. Table 1 lists the parameter values of the SEIR model and the mean value of the parameter distributions for the ABMC simulation. Note that, in general, the means of the lifetime distributions in the ABMC are simply the reciprocals of the rates used in System (6). However, so that infection event times always depend on the current size of the susceptible population *S*(*t*) (similar to what is assumed in System (6)), the infection time distribution mean is continuously updated. Figure 2, shows the progression of an individual through the epidemic.

## Results and discussion

### Basic reproductive ratio

The basic reproductive ratio *R*_0_ is defined as the number of newly infectious individuals produced by one infectious individual in a totally susceptible population. For a description of different methods for calculating *R*_0_ see [22]. For System (6),

(7) $$R_{0}=\frac{\beta N}{\gamma }\;.$$

where *N* = *S*_0_ is the total population size of susceptibles at time zero. Note that the calculation of *R*_0_ is independent of *f*(*I*,*p*).

### Comparison of media functions

**Figure 3:**
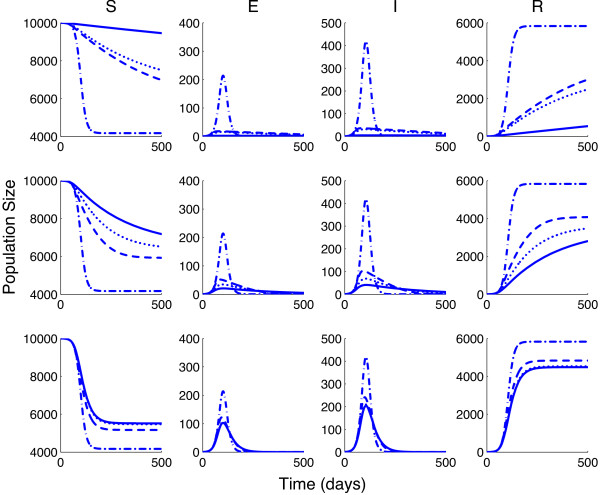
**Epidemic curves using the different values for each media function.** Functions (1) (dotted line), (2) (dashed line), and (3) (solid line) are shown when *p*_3_ = 10 (top), 100 (middle), 1000 (bottom). *p*_1_ and *p*_2_ are determined by equations (4) and (5) with *γ* = 1/4 and *Ic* = 300. For comparison, the standard SEIR model 6 with no media effect is also shown (dash-dotted line).

Figure 3 plots System (6) using Media Functions (1)-(3) for *p*_3_ = 10, 100, and 1000 (solid line), with and *p*_1_ (dotted line) and *p*_2_ (dashed line) determined by Equations (4) and (5), and *γ* = 1/4 and *I*_*c*_ = 300. For comparison, the standard SEIR model with no media effect is also shown (dash-dotted line). This figure demonstrates that mass media will affect the epidemic curve. It also demonstrates that the epidemic curve varies depending on the media function used.

**Figure 4:**
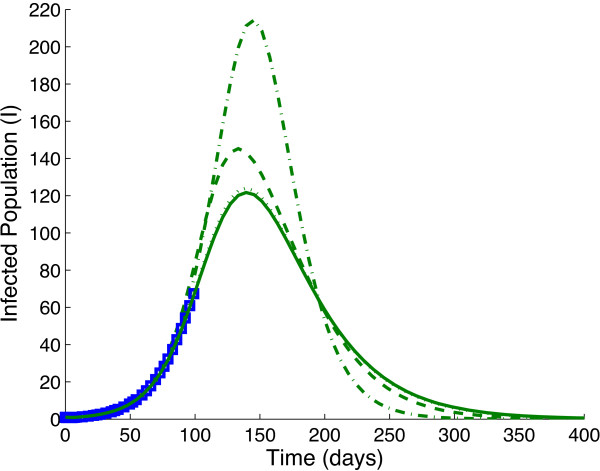
**Data and fitted models.** The epidemic curves are shown resulting from fitting System (6) with Media Functions (1)-(3) (dotted line, dashed line, solid line) and no media (dash-dotted line) to 100 days of epidemic data (boxes) generated using Media Function (3) with *p*_3_ = 1000 and *γ* and *σ* from Table 1.

For a further comparison of the media functions, we determine the values of the basic reproductive ratio *R*_0_ and the media constant *p* of *f*(*I*,*p*) based on epidemic data generated from our models. For an example, Table 2 lists the values of *β* and *p* determined through a fitting routine (in MATLAB - various routines were used and similar results were obtained) over the first 100 days of an epidemic, with data generated using Media Function (3) with *σ* and *γ* values listed in Table 1 and *p*_3_ = 1000. Figure 4 plots the resulting epidemic curves of System (6) using Media Functions (1)-(3) (dotted, dashed and solid lines) and no media (dash-dotted line). Table 2 and Figure 4 demonstrate that even though all of the models have a very similar shape and basic reproductive ratio *R*_0_ at the beginning of the epidemic, the resulting epidemic curves can still vary drastically depending on the media function chosen. Similar observations were made using different numbers of days of data used to fit *β* and *p*, and different Media Functions to generate the data (not shown). Therefore, it can be concluded that key epidemic measurements – peak number of infections, time of peak, end of epidemic, and total number of infections – will also vary depending on the Media Function chosen.

**Table 2:** **Model and fitted parameters *β * and** ***p***_***i***_

**Model**	***β***	***p***_***i***_	***R***_**0**_
no media	3.29 × 10^-5^		1.32
(1)	3.33 × 10^-5^	0.00365	1.33
(2)	3.32 × 10^-5^	5.2 × 10^-5^	1.33
(3)	3.33 × 10^-5^	1000	1.33

Based on 100 days of data generated using System (6) with Media Function (3) with *σ* and *γ* values listed in Table 1 and *p*_3_ = 1000.

**Figure 5:**
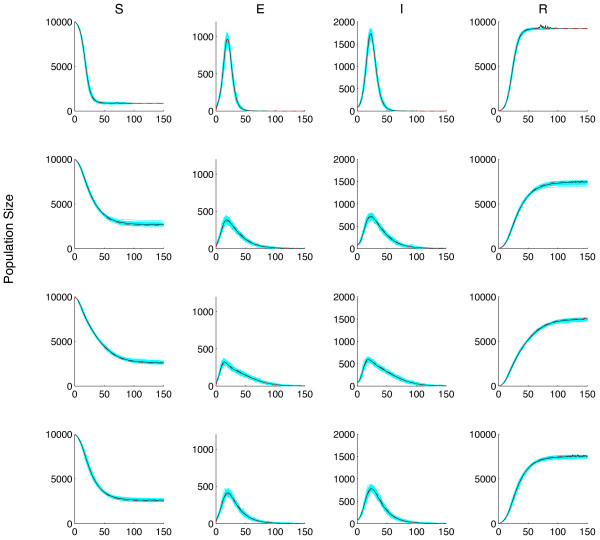
**Epidemic curves for different media functions.** The epidemic curves are shown for Model (6) (dashed line) and the ABMC simulation (gray lines). There are 100 simulations plotted for each. In each panel, we also show the mean of the simulations (sold line). The first row has no media effect and rows (2-4) correspond to Functions (1)-(3). Here, *I*_*c*_ = 300 and *p*_3_ = 1000, $\gamma =\frac{1}{4}$.

System (6) is useful in describing the mean behaviour of an epidemic, but it is unable to provide estimates of variation of important public health measures. A stochastic simulation lends itself well to demonstrating variation within an epidemic. Here, we employ an Agent-Based Monte Carlo (ABMC) simulation. Figure 5 shows 100 simulation runs of the ABMC simulation when no media is involved (top), and when Media Functions (1)-(3) are used (second to bottom rows). Each simulation run (gray line) is shown, as well the mean of the simulation runs (solid line) and solution of the System (6) with the corresponding media functions (dashed line). Note that the mean (solid line) and solution to System (6) are in agreement. This figure demonstrates that variability in the key epidemic measurement occurs. Table 3 lists the mean and standard error of each epidemic measurement when no media is considered (section a), and Media Functions (1)-(3) are included in the simulation (Sections b-d). In each section we list the simulation mean and standard error (top row), and the solution of System (6).

**Table 3:** Key epidemic measurements for the SEIR model with (a) no media, (b) media function (1), (c) media function (2), and (d) media function (3)

**Model**	**Peak Magnitude**	**Peak time**	**Epidemic end**	**Total**
	**(E+I)**	**(days)**	**(days)**	**(I)**
(a)	2676.1	21.155	76.6065	9254.8
	2623.9±204.09	21.005±19.9	77.1863±15.59	9542.4±255.37
(b)	1061.5	21.7841	162.3647	7537.5
	1095.6±46.35	21.001±19.99	168.077±31.24	7578.13±460.95
(c)	858.176	18.544	176.8229	7557.9
	902.15±78.85	17.7±15.99	177.07±23.6	7622.2±328.8
(d)	1144.7	23.5169	154.3623	7586.0
	1175.9±175.09	22.01±20.99	157.07±28.005	7643.9±229.2

The results of system 6 and the ABMC are listed in the top and bottom row of each section respectively with *p*_3_ = 1000, *p*_1_ and *p*_2_ determined using equations (4) and (5), and *γ*= 1/4 and *I*_c_ = 300. 500 simulation runs are used for the ABMC calculations.

### Sensitivity analysis

Within public health settings, a goal is to identify key characteristics of an epidemic that drive the infection with the hope of determining public health measures that can be implemented so that control or eradication of the pathogen can be achieved. By performing sensitivity analysis on System (6) with Equations (1)-(3), parameters that most affect epidemic outcomes for each media function can be identified, informing policy makers so that appropriate public health measures can be put into place. To conduct the sensitivity analysis, we use Latin Hypercube Sampling (LHS) and partial rank correlation coefficients (PRCC) [23].

**Figure 6:**
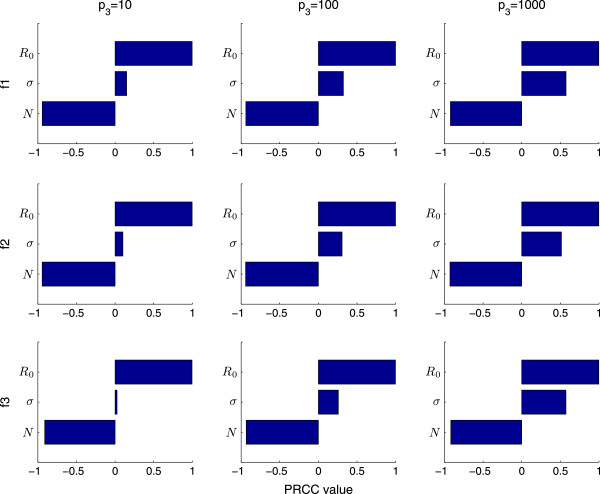
**LHS-PRCC results for** ***I***_***c***_** = 300 for peak magnitude.** This sensitivity analysis is done with 1000 bins. The rows correspond to Function (1), Function (2) and Function (3), respectively. The columns of this PRCC figure correspond to *p*_3_ = 10, *p*_3_ = 100 and *p*_3_ = 1000. Here *I*_*c*_ = 300 and $\gamma =\frac{1}{4}$.

**Figure 7:**
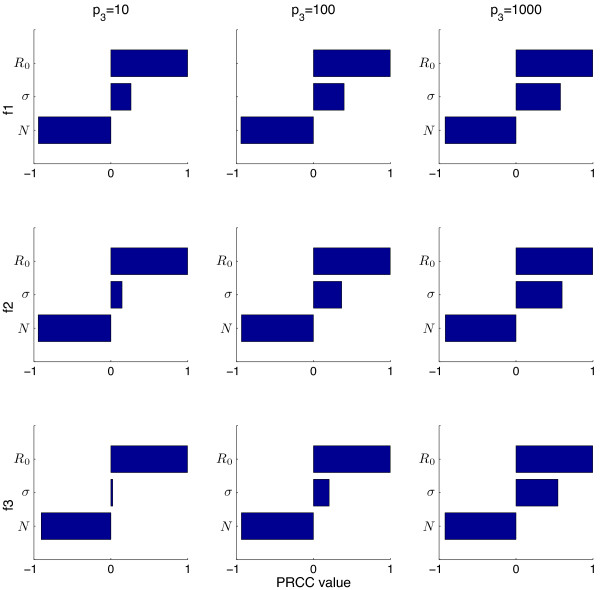
**LHS-PRCC results for** ***I***_***c***_** = 1000.** This sensitivity analysis is done with 1000 bins. The rows correspond to Function (1), Function (2) and Function (3), respectively. The columns of this PRCC figure correspond to *p*_3_ = 10, *p*_3_ = 100 and *p*_3_ = 1000.

We first conduct the sensitivity analysis with *I*_*c*_, *p*_3_ and *γ* constant to directly compare the media functions. Figures 6 and 7 show the PRCC values determined for peak magnitude when *I*_*c*_ = 300 (Figure 6) and 1000 (Figure 7) for Functions (1)-(3) (top to bottom) when *p*_3_ = 10, 100 and 1000 (left, middle and right columns respectively). These figures demonstrate that for each set of parameters the PRCC value is similar. This is true for each key epidemic measurement - the PRCC values are similar between different values of *p*_3_ and *I*_*c*_. However the PRCC values between key measurements are different i.e. the PRCC values are different if comparing peak magnitude to epidemic end time (not shown).

**Figure 8:**
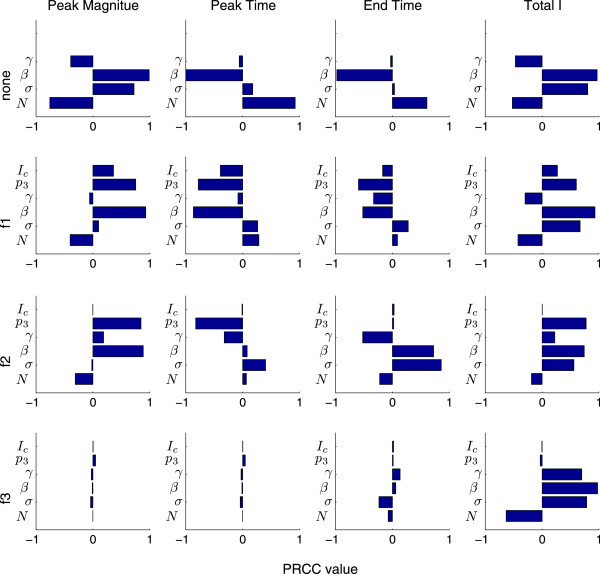
**PRCC for system (6).** Results are shown for peak magnitude, peak time, epidemic end time, and total number of infections (left to right) when no mass media function is considered, and functions (1)-(3) are used (top to bottom). Here, *p*_3_ = 100 and *p*_1_ and *p*_2_ are determined by Equations (4) and (5).

For further sensitivity analysis, all parameters are allowed to vary. Figure 8 shows the result of the sensitivity analysis for all four outcomes key to public health: peak number of infections, peak time, epidemic end time, and total number of infections. The results demonstrate that the SEIR model is more (or less) sensitive to certain parameters depending on what Mass Media Function (1)-(3) is used. For example, changes in *β* have a large effect on peak magnitude when Function (1) is used, but it has little to no effect when Functions (2-3) represent the effects of media. Variable *p*_3_ also has a large effect on peak magnitude when media is represented by Function (1). It has a similar effect when Function (2) is used, but it has no effect on the system utilizing Function (3).

The sensitivity analysis indicates that models that include mass media influence will greatly depend on different parameters, depending on the media function chosen. This makes it very difficult for policy makers to determine an effective public health intervention strategy. This also explains the very different epidemic curves produced by System (6) when different media functions are employed, notwithstanding the similarities in the media functions when plotted at a specific level of media.

## Conclusion

Technology and media play an increasing role in daily life. Mass media that is transmitted via technological media can therefore be used to inform the public during pandemics and epidemics. An understanding of the effects of media during an epidemic or pandemic threat can aid in the development of public health policy. Of particular interest to public health are the effects of media on key epidemic measurements - peak magnitude of infection, time of peak, end of epidemic, and total number of infections.

Mathematical modelling has been used to study the effect of media on epidemics by employing functions in the transmission terms of mathematical models [6-14]. A survey of the literature identified three functions that have been utilized to represent media in disease modelling [6-14]. We have conducted a comparison of these functions to determine the effects of media function on key epidemic measurement and variability within these measurements. We first demonstrated that by including mass media in System (6) the peak magnitude of the epidemic and the total number of infections would decrease. We also determined that the time to peak and the end of the epidemic would also occur earlier. However, we also demonstrated that, although the functions are similar in shape and magnitude, the resulting epidemic curve can be quite different (Figure 5). Therefore, the key epidemic measurements that are used to inform public health policy will be different. Furthermore, we demonstrated that variability in the key epidemic measurements also depends on the media function chosen (Figure 5 and Table 3).

A sensitivity analysis on System (6) with the different media functions was also conducted. Obtained from this analysis was the insight that some parameters are important for some outcomes and not for others. We can conclude that due to the different fixed functions resulting in very different epidemic behaviours, there is no clear control strategy present. Also, as a reult of the different behaviours from the different media functions, we are unsure as to which is the best function to use to model mass media. This suggests that a function representing media may not be the best course for modelling the effects of media during an epidemic. We suggest that perhaps, a separate model compartment representing media reports, such as those incorporated into surveillance data [24] could be used. Development of such a model is a course for future work.

Mass media reports can affect social behaviour, that ultimately, affects transmission of disease. However, an individual’s response to a media stimulus will wane over time [13,25-29]. Models that employ a media function such as those studied here are difficult to modify to involve a waning response to a media stimulus over the age of an epidemic. This can be incorporated easily into a model whereby media is represented as a model compartment. A study of the effects of ‘waning media’ is a course for future work.

The current study has employed a simple SEIR model that includes three parameters. This model implicitly assumes that individuals mix at random in the population, the age and sex of individuals is unimportant, and that the population size remains constant over the epidemic. These assumptions are not true reflections of reality. An interesting direction for future work is to consider the effects of mass media reports on individual decision making strategies, and study how mass media can impact the contact structure of a population network [30].

The simple SEIR model employed in this study was used to study one season of influenza. Typically, seasonal forcing is used to model multiple seasons of influenza. Inclusion of mass media reports and media waning in a model of multiple seasons with seasonal forcing is an interesting direction for study.

## Competing interests

The authors declare that they have no competing interests.

## Authors’ contributions

MSC performed the literature review. JMH and MSC developed the models, conducted the analysis and wrote the paper. Both authors read and approved the final manuscript.

## Pre-publication history

The pre-publication history for this paper can be accessed here:

http://www.biomedcentral.com/1471-2458/14/376/prepub
